# Adjunctive Treatments Reduce Ischemia-Reperfusion Injury in Breast Reconstruction After Mastectomy: A Systematic Review

**DOI:** 10.7759/cureus.117393

**Published:** 2026-10-03

**Authors:** Marina M Georgies, Iyawnna Hazzard, Manya Bali, Ethan A Tabaie, Jose Puglisi, Eldo Frezza

**Affiliations:** 1 Department of Research, California Northstate University College of Medicine, Elk Grove, USA

**Keywords:** adjunctive therapy, breast reconstruction, flap loss, ischemia-reperfusion injury, mastectomy

## Abstract

Ischemia-reperfusion injury (IRI) is a common postoperative outcome of free flap surgery in breast reconstruction post-mastectomy. Various approaches to limiting IRI have been investigated. The purpose of this review is to evaluate studies that compare ischemia-reducing therapies in breast reconstruction patients.

PubMed and Embase databases were screened using a search algorithm. Patient demographics and study details, including ischemia-reducing intervention agents and postoperative outcomes, such as flap loss, thrombosis, systemic response, and vascular response, were extracted for evaluation.

Ten studies were included in this review, with data on 1178 flaps in 935 female patients. Postoperative infusion of IV arginine resulted in greater blood flow to the distal flap (p = 0.04). Intervention with IV methylprednisolone yielded significantly reduced rates of partial flap necrosis (PFN) (p = 0.009), significantly decreased postoperative levels of IL-1β (p = 0.0004), IL-10 (p = 0.00044), and TNF (p = 0.00011), and shorter length of hospital stay (p = 0.00001).

Many adjunctive therapies are associated with lower rates of IRI in breast reconstruction post-mastectomy. Of these therapies, postoperative IV arginine resulted in greater blood flow to the distal flap, while perioperative IV methylprednisolone resulted in a significant reduction in PFN and other markers of IRI. Future studies should focus on the effects of steroid and/or vasodilator administration on IRI and seek to establish lasting protective effects.

## Introduction and background

Breast cancer is the second leading cause of cancer and cancer death in females worldwide [1]. It is typically treated with surgical mastectomy, radiation, immunotherapy, chemotherapy, hormone-receptor blockers, and bone-modifying reagents [2]. Surgical mastectomy techniques include simple mastectomy, nipple-sparing mastectomy, and many others [3]. Due to harsh deformities [4] and psychological problems surrounding body image [5] from mastectomy, nearly one-third of women undergo breast reconstruction following mastectomy [6]. Breast reconstruction may use implants or autologous tissue, with free-flap reconstruction involving the transfer of a patient’s own tissue from one part of the body to the breast. During free-flap reconstruction, the blood vessels supplying the transferred tissue are divided during tissue harvest and then surgically connected to recipient blood vessels in the chest using microsurgical techniques to restore blood flow. Although restoration of blood flow is essential for flap survival, the period of interrupted blood supply and subsequent restoration of circulation can result in ischemia-reperfusion injury (IRI) [7].

Initially in IRI, as a result of detachment of breast tissue during mastectomy, regional blood supply is temporarily halted, inducing oxygen deprivation and reactive oxygen species formation. Then, following the establishment of artery and vein anastomosis during reconstruction with free-flap tissue, reperfusion triggers a sudden influx of blood and oxygen, leading to an inflammatory response, oxidative stress, and microvascular damage [8]. Mitochondrial dysfunction, endothelial dysfunction, and antioxidant enzymes such as superoxide dismutase (SOD) are all potential markers of reperfusion injury [9].

Early management of IRI is essential for flap survival. Thus, there have been efforts to add an ischemia-reducing adjuvant, such as arginine [10], corticosteroids [8], sevoflurane [11], or others, during breast reconstruction surgery to reduce the occurrence and/or extent of IRI post-mastectomy. To our knowledge, there is minimal literature on adjunctive therapies used in this clinical setting. Therefore, the primary objective of this paper is to determine which adjunctive interventions, if any, are effective in reducing IRI in breast reconstruction post-mastectomy.

## Review

Methods

Search Strategy

A search following guidelines established by the Preferred Reporting Items for Systematic Reviews and Meta-Analyses (PRISMA) was performed in two major biomedical databases, PubMed and Embase, which were selected to provide sufficiently broad coverage of the medical and surgical literature relevant to breast reconstruction and IRI. The query was performed utilizing the Boolean search phrase "Mammaplasty"[MeSH Terms] OR "Breast Reconstruction" OR "Mammaplasty" OR "Mammoplasty") AND ("Reperfusion Injury"[MeSH Terms] OR "Injury, Ischemia-Reperfusion" OR "Injury, Reperfusion" OR "Ischemia-Reperfusion Injury" OR "Reperfusion Damage" OR "Reperfusion Injury"). There were no restrictions set for the search. Inclusion criteria were structured according to the Population, Intervention, Comparison, and Outcome (PICO) framework. Studies were eligible for inclusion if they evaluated human patients undergoing autologous free-flap breast reconstruction following mastectomy and investigated an adjunctive intervention intended to reduce or mitigate IRI, or evaluated an ischemia-reperfusion-related response associated with free-flap reconstruction. Studies were eligible if they included a control group receiving standard care, placebo, no adjunctive intervention, or an alternative intervention, or if they were non-comparative studies that provided clinically relevant information regarding IRI. Eligible studies were required to report at least one IRI-related outcome, including flap loss or necrosis, microvascular thrombosis, inflammatory or oxidative stress markers, vascular response, flap perfusion, or other postoperative measures of IRI. Studies were excluded if they were conducted in animals or non-human models, did not involve breast reconstruction following mastectomy, did not evaluate autologous free-flap reconstruction, or did not report an ischemia-reperfusion-related outcome. Case reports, review articles, conference abstracts, expert opinions, letters to the editor, and non-English-language articles were also excluded. This study was registered on PROSPERO (ID: 557464).

Study Selection

Two independent reviewers (MG and IH) reviewed studies for eligibility criteria from the initial database search. A third senior author was available for any disputes. All included articles underwent a rigorous reference search to determine whether additional studies could be added to the systematic review.

Data Extraction

Study variables extracted from each article included authors, publication title, publication year, study design, country, number of patients, mean age, mean BMI, number of flaps, flap source, mean total flap weight, risk factors, length of hospital stay, post-operative complications, ischemia-reducing agents, ischemia period, evidence of IRI (flap loss, thrombosis, inflammatory markers, oxidative stress markers, and vascular response markers), and other post-operative outcomes (resolution of IRI, etc.). All extracted data were compiled for analysis using Microsoft Word (Microsoft Office 2011; Microsoft® Corp., Redmond, WA, USA).

Quality Assessment and Risk of Bias (ROB)

The quality of studies was assessed using the Methodological Index for Non-Randomized Studies (MINORS) checklist. The MINORS items are scored 0 (not reported), 1 (reported but inadequate), or 2 (reported and adequate), with a maximum possible score of 16 for non-comparative studies (from eight categories) and 24 for comparative studies (from 12 categories). Two authors scored each article in the systematic review. Each author scored the article individually before reviewing their scores, and any discrepancies were resolved by re-reviewing the articles until a unanimous consensus was reached. The ROB was determined based on the overall MINORS score. The ROB was considered high if the MINORS score was 0 to 8 (0 to 16 for comparative studies), moderate if the MINORS score was 9 to 12 (16 to 20 for comparative studies), and low if the MINORS score was 13 to 16 (21 to 24 for comparative studies).

Statistical Analysis

Statistics (mean, percentage, standard deviation, range, and median) are reported in this review when applicable and available.

Results

Article Selection Process

Thirty studies were identified from a search of Embase and PubMed databases, of which 11 duplicates were removed. The remaining 19 studies underwent full title and abstract review, of which five were removed based on our predetermined exclusion criteria. The remaining 14 studies underwent full-text review, and four of these studies were excluded as they did not fit our predetermined inclusion criteria. The remaining 10 were included in this systematic review. The PRISMA flow diagram depicting our search strategy and method of selecting articles is depicted in Figure 1.

**Figure 1:**
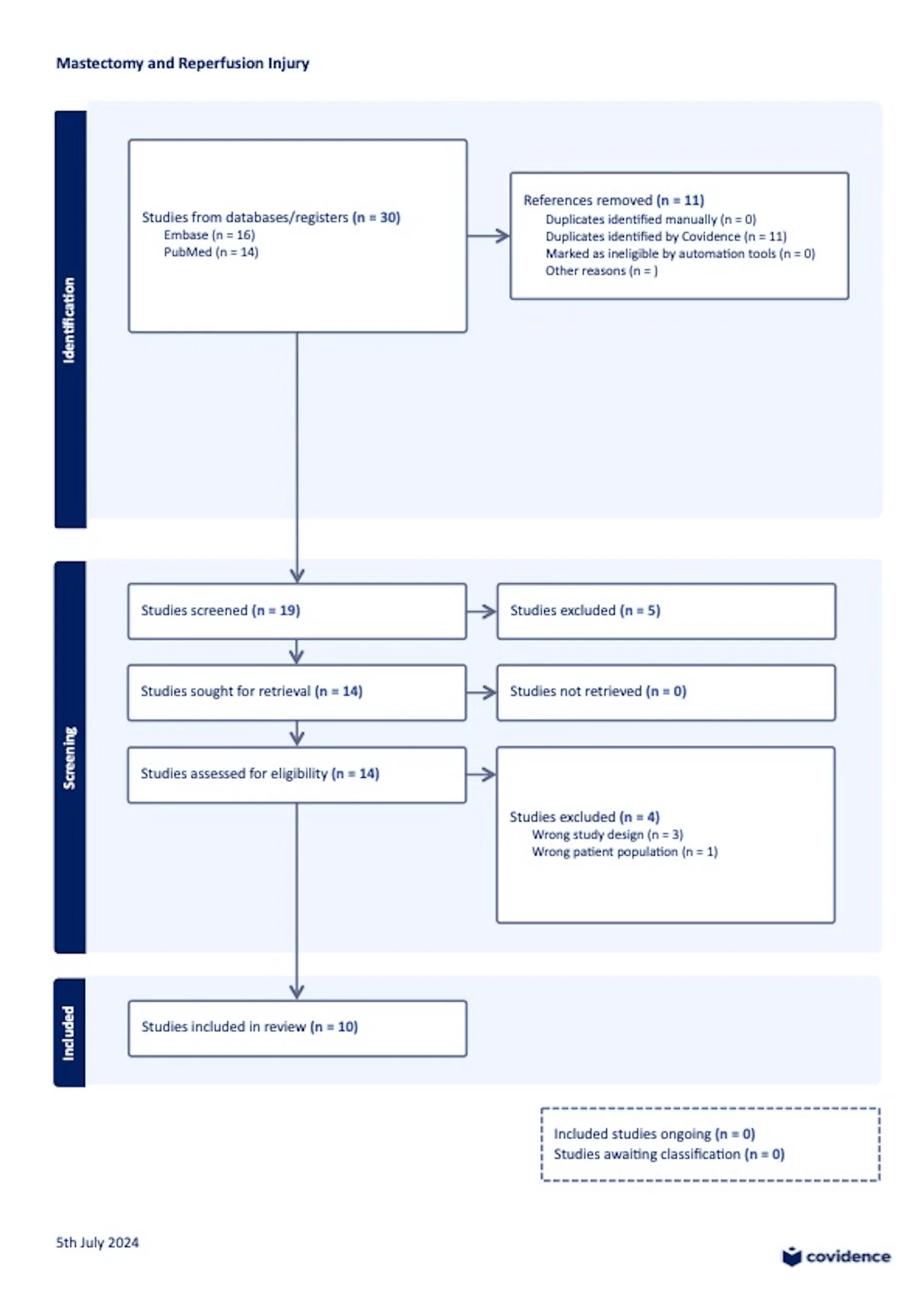
PRISMA (Preferred Reporting Items for Systematic Reviews and Meta-Analyses) Flowchart

Methodological Index and ROB Assessment

The mean ± standard deviation (range) of the MINORS score for comparative studies discussing IRI in breast reconstruction after mastectomy was 19.75 ± 3.69. For noncomparative studies, the mean MINORS score was 10.5 ± 2.12. Overall, the ROB was low for five studies [8,10-13], moderate for three studies [7,14,15], and high for two studies [16,17]. The total MINORS score is reported for each study in Figure 2.

**Figure 2:**
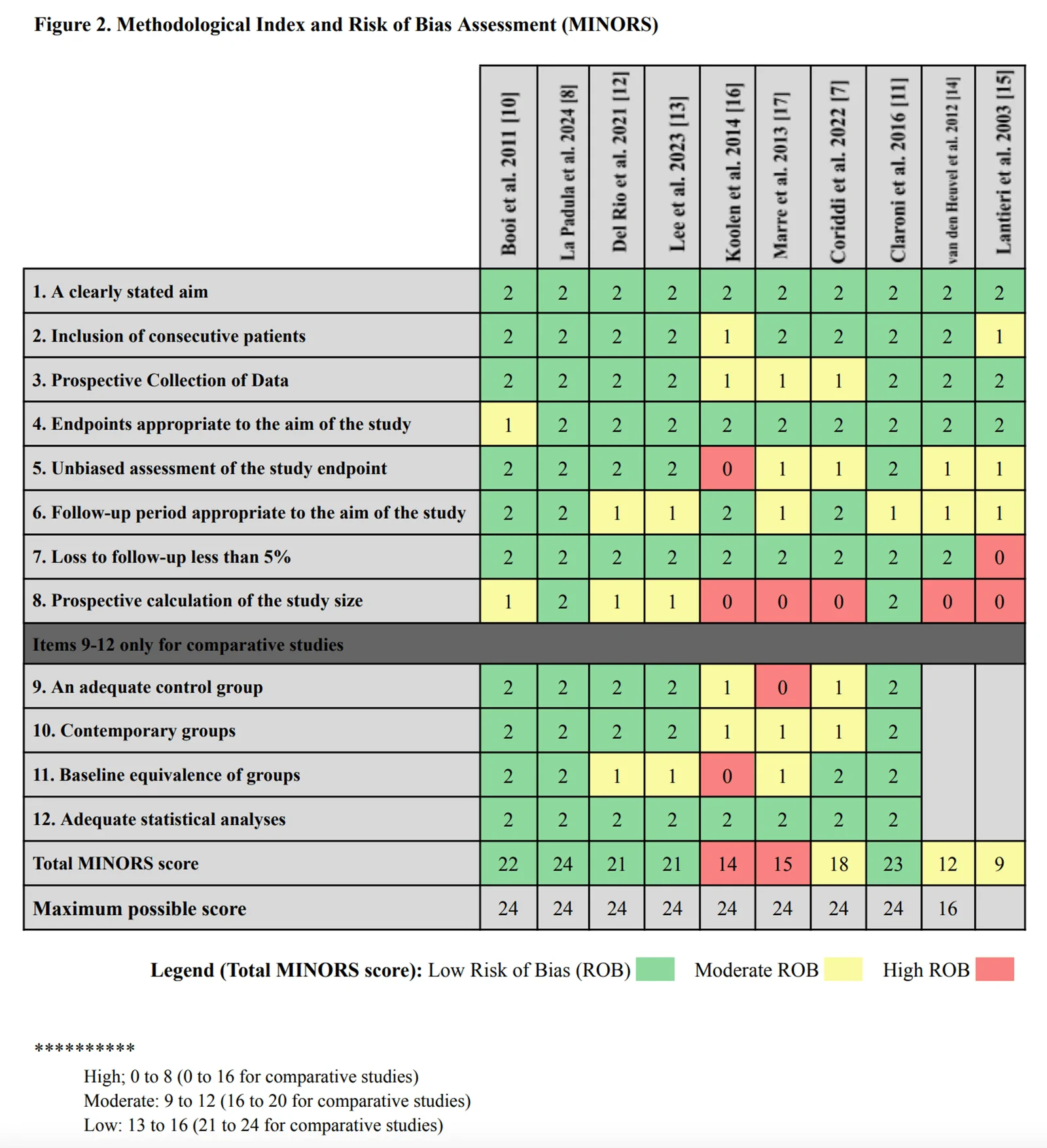
Methodological Index and Risk of Bias Assessment (MINORS)

Study Characteristics, Patient Demographic Information, and Flap Details

The demographic data reported in this study include the author of each study, study title, publication year, study type, level of evidence (LOE), country, number of patients, age, BMI, number of flaps, flap source, and total flap weight, all of which are summarized in Table 1. The 10 studies [7,8,10-17] included in this review were published between 2003 and 2024. Three studies had a retrospective design [7,16,17], and seven had a prospective design [8,10-15]. The LOE was II in five studies [8,10-13], III in one study [14], and IV in four studies [7,15-17]. Across all 10 included studies, a total of 935 patients (all female) were identified, with mean ages ranging from 43 ± 3 to 55 ± 8.6 years and mean BMIs ranging from 22.2 ± 2.7 to 30.1 kg/m². A total of 1178 flaps from various sources were identified, with mean total weights ranging from 504 ± 174 to 999 ± 133 grams.

**Table 1:** Study Details, Patient Demographics, and Flap Details LOE, level of evidence; RCT, randomized control trial; TG, treatment group; CG, control group; NR, not reported; TRAM, transverse rectus abdominis myocutaneous; fTRAM, free TRAM; MS-TRAM, muscle sparing TRAM; msfTRAM, muscle-sparing free TRAM; DIEP, deep inferior epigastric perforator; SIEA, superior inferior epigastric artery; SGAP, superior gluteal artery perforator; DUG, diagonal upper gracilis; PAP, profunda artery perforator; CVM, other cardiovascular medications

Author	Title	Publication Year	Study Design	LOE	Country	Number of Patients	Age (years)	BMI (kg/m^2^)	Number of Flaps	Flap Source	Total Flap Weight (g)
Booi et al. [10]	Arginine improves microcirculation in the free transverse rectus abdominis myocutaneous flap after breast reconstruction: a randomized, double-blind clinical trial.	2011	Prospective RCT	II	Netherlands	Total: 18; TG: 8; CG: 10	TG: 47 ± 3; CG: 43 ± 3	TG: 27 ± 1; CG: 24 ± 1	18	TRAM	TG: 809 ± 111; CG: 999 ± 133
La Padula et al. [8]	Effect of corticosteroids on ischemia-reperfusion injury of deep inferior epigastric perforator flap after re-exploration for anastomosis thrombosis: A prospective randomized trial.	2024	Prospective RCT	II	Italy	Total: 46; TG: 23; CG: 23	TG: 54 ± 9.3; CG: 55 ± 8.6	TG: 27.96 ± 3.56; CG: 27.38 ± 3.51	46	DIEP	TG: 570 ± 95.43; CG: 545 ± 74.39
Del Rio et al. [12]	Effect of intravenous lidocaine on ischemia-reperfusion injury in DIEP microsurgical breast reconstruction. A prospective double-blind randomized controlled clinical trial.	2021	Prospective RCT	II	Spain	Total: 24; TG: 12; CG: 12	TG: 47.75; CG: 48.41	TG: 29.3; CG: 30.1	24	DIEP	NR
Lee et al. [13]	Effect of sevoflurane-remifentanil and propofol-remifentanil anesthesia on glycocalyx shedding during deep inferior epigastric perforator flap breast reconstruction: a prospective randomized, controlled trial.	2023	Prospective RCT	II	Korea	Total: 50; Sevoflurane: 25; Propofol: 25	Sevoflurane: 49 ± 7; Propofol: 48 ± 9	Sevoflurane: 24.95 ± 4.42; Propofol: 23.82 ± 3.62	50	DIEP	Sevoflurane: 518 ± 73; Propofol: 504 ± 174
Koolen et al. [16]	Effects of statins on ischemia-reperfusion complications in breast free flaps.	2014	Retrospective Chart Review	IV	USA	Total: 482; CG: 549; Statin: 45; CVM: 70; Statin+CVM: 38	48.1 ± 8.1	27.5 ± 5.1	702	DIEP (646); SIEA (15); SGAP (32); msfTRAM (3); fTRAM (4)	NR
Marre and Hontanilla [17]	Increments in ischemia time induce microvascular complications in the DIEP flap for breast reconstruction.	2013	Retrospective Chart Review	IV	Spain	Total: 182	49 ± 8.8	25.7 ± 3.8	200	DIEP	639.4 ± 257.3
Coriddi et al. [7]	Management of postoperative microvascular compromise and ischemia-reperfusion injury in breast reconstruction using autologous tissue transfer: Retrospective review of 2103 flaps.	2022	Retrospective Chart Review	IV	USA	Total: 44	49 ± 8.0	29.0 ± 5.1	47	DIEP (31); MS-TRAM (12); DUG (2); SGAP (1); PAP (1)	NR
Claroni et al. [11]	Protective effect of sevoflurane preconditioning on ischemia-reperfusion injury in patients undergoing reconstructive plastic surgery with microsurgical flap, a randomized controlled trial.	2016	Prospective RCT	II	Italy	Total: 54; Sevoflurane: 27; Propofol: 27	Sevoflurane: 50 ± 7.5; Propofol: 52.7 ± 7.8	Sevoflurane: 22.2 ± 2.7; Propofol: 23.1 ± 2.9	NR	DIEP	NR
van den Heuvel et al. [14]	The role of antioxidants in ischemia-reperfusion in a human DIEP flap model.	2012	Prospective Cohort Study	III	Netherlands	Total: 15	NR	NR	17	DIEP	NR
Lantieri et al. [15]	Tissue and plasma levels of endothelin in free flaps.	2003	Pre-post Study	IV	France	Total: 20	NR	NR	20	TRAM DIEP	NR

Risk Factors, Length of Hospital Stay, and Postoperative Complications

The number of patients with predetermined risk factors, length of hospital stay, and postoperative complications excluding IRI are summarized in Table 2. Risk factors include radiation therapy, chemotherapy, smoking (or history of smoking), hypertension, diabetes mellitus, hyperlipidemia, etc. Five studies reported patients who underwent radiation or chemotherapy prior to the conduction of the study [7,8,10,13,16,17]. Smoking (or smoking history) was a common risk factor, reported in five studies [7,10,13,16,17]. Hypertension was the most commonly reported risk factor, cited in six studies [7,8,11,13,16,17]. Of these six, one study [16] found that hypertension was significantly higher in patients on CVMs (cardiovascular medications) and in patients on both statins and CVMs when compared to the non-medication group (p < 0.001). Diabetes was cited in five studies [7,8,13,16,17], one of which [16] reported a significantly higher prevalence of diabetes in patients on both CVMs and statins (p < 0.001). Other comorbidities included depression and asthma [8], blood disorders and previous thrombotic events [16], and hyperlipidemia/dyslipidemia [7,11,16]. One study [16] reported a significantly higher prevalence of hyperlipidemia in patients taking both statins and CVMs (p = 0.001).

**Table 2:** Risk Factors, Length of Hospital Stay, and Postoperative Complications IRI, ischemia-reperfusion injury; TG, treatment group; CG, control group; CVM, cardiovascular medication

Author	Number of Patients	Number of Patients with Risk Factor(s)						Length of Hospital Stay (days)	Postoperative Complications (not including IRI)	
		Radiation	Chemotherapy	Smoking	HTN	Diabetes	Other		No Complications	Other Complication
Booi et al. [10]	18; TG: 8, CG: 10	TG: 2; CG: 6; p = 0.15	TG: 4; CG: 9; p = 0.09	TG: 2; CG: 3; p = 0.59	-	-	-	TG: 7 ± 0; CG: 7 ± 0	TG: 6; CG: 5	-
La Padula et al. [8]	46; TG: 23, CG: 23	TG: 19; CG: 20; p = 0.2	TG: 19; CG: 20; p = 0.5	-	TG: 2; CG: 1; p = 0.2	TG: 1; CG: 2; p = 0.2	Depression: TG: 3, CG: 4; Asthma: TG: 1, CG: 2	TG: 11.13 ± 0.38; CG: 15.47 ± 1.27; p = 0.00001	-	-
Del Rio et al. [12]	24; TG: 12, CG: 12	-	-	-	-	-	-	-	-	-
Lee et al. [13]	50; Sevoflurane: 25, Propofol: 25	Post-op Sevoflurane: 9, Propofol: 7	Pre-op Sevoflurane: 7, Propofol: 6, p = 0.747; Post-op Sevoflurane: 4, Propofol: 3	Ex-smokers: Sevoflurane: 0, Propofol: 3	Sevoflurane: 2, Propofol: 3, p > 0.999	Sevoflurane: 1, Propofol: 0, p > 0.999	-	Sevoflurane: 8.4 ± 2.5, Propofol: 7.4 ± 1.0	-	Reoperation Sevoflurane: 4, Propofol: 1; Flap detachment: Sevoflurane: 1, Propofol: 0; Hematoma: Sevoflurane: 3, Propofol: 1; Wound dehiscence: Sevoflurane: 1, Propofol: 1; Venous congestion: Sevoflurane: 2, Propofol: 1
Koolen et al. [16]	482 medication use per flap: Statin: 45, CVM: 70, Statin + CVM: 38, CG: 549	-	-	Statin: 2, CVM: 0, Statin + CVM: 0, CG: 32, p = 0.083	Statin: 5, CVM: 34, Statin + CVM: 13, CG: 17, p < 0.001 (compared to CG)	Statin: 0, CVM: 4, Statin + CVM: 5, CG: 11, p < 0.001 (compared to CG)	Blood Disorder: Statin: 1, CVM: 0, Statin + CVM: 0, CG: 6; Previous Thrombotic Event: Statin: 0, CVM: 0, Statin + CVM: 0, CG: 3; Hyperlipidemia: Statin: 0, CVM: 0, Statin + CVM: 1, CG: 0, p = 0.001 (compared to CG)	-	-	Wound Dehiscence Breast: Statin: 4 (p = 0.017), CVM: 7 (p = 0.001), Statin + CVM: 1, CG: 14; Wound Dehiscence Abdomen: Statin: 6, CVM: 19 (p < 0.001), Statin + CVM: 2, CG: 35; Overall Complication Rate: Statin: 21 (p = 0.037), CVM: 32 (p = 0.017), Statin + CVM: 14, CG: 173
Marre and Hontanilla [17]	182	Pre-op: 93, Post-op: 27	Pre-op: 118, Post-op: 42	141	161	182	-	-	-	-
Coriddi et al. [7]	44	Pre-op: 20, Post-op: 4	Pre-op: 22, Post-op: 13	Ex-smokers: 10, Current smokers: 2	5	1	Hyperlipidemia: 6	-	-	-
Claroni et al. [11]	54; Sevoflurane: 27, Propofol: 27	-	-	-	Sevoflurane: 1, Propofol: 1	-	Dyslipidemia: Sevoflurane: 2, Propofol: 0	-	-	-
van den Heuvel et al. [14]	15	-	-	-	-	-	-	-	-	-
Lantieri et al. [15]	20	-	-	-	-	-	-	-	-	-

Three studies [8,10,13] reported the average length of hospital stay following breast reconstruction surgery, and one study [8] found that the treatment group (given methylprednisolone) had a significantly shorter postoperative stay (p = 0.00001). One study [10] found similar instances of complication-free outcomes between the control and treatment groups (given arginine). Two other studies [13,16] reported various postoperative complications besides IRI (IRI is covered in greater detail later in this paper). Of these outcomes, one study [16] found significant differences in complication rates among groups, with a higher prevalence of complications occurring in certain medication groups compared with the control. Higher rates of wound dehiscence in the breast occurred in the statin group (p = 0.017) and CVM group (p = 0.001); higher rates of wound dehiscence in the abdomen occurred in the CVM group (p < 0.001); and higher overall complication rates occurred in the statin group (p = 0.037) and the CVM group (p = 0.017) [16].

Ischemia-Reducing Agent and IRI

The number of patients in each study, ischemia-reducing agent, ischemia period, type of flap loss (total flap loss - TFL, partial flap loss - PFL, and partial flap necrosis - PFN), type of thrombosis (arterial, venous, and unspecified), systemic response markers, and vascular response markers are summarized in Table 3. Mean ischemia period during breast reconstruction was reported in five studies [8,10,12,15,17] and ranged from 44 ± 3 minutes [10] to 177 minutes [17]. Three studies that utilized interventions with methylprednisolone [8], arginine [10], or lidocaine [12] found no significant difference in ischemia time between study groups. One study [17] retrospectively grouped subjects into quartiles based on ischemia time, reporting significantly higher rates of postoperative thrombosis in the upper two quartiles (p = 0.02).

**Table 3:** Ischemia-Reducing Agent and Ischemia Reperfusion Injury TG, treatment group; CG, control group; TRAM, transverse rectus abdominis myocutaneous; fTRAM, free TRAM; ms fTRAM, muscle-sparing fTRAM; DIEP, deep inferior epigastric perforator; SGAP, superior gluteal artery perforator; SIEA, superior inferior epigastric artery; DUG, diagonal upper gracilis; PAP, profunda artery perforator; TFL, total flap loss; PFL, partial flap loss; PFN, partial flap necrosis; BASAL, Before surgery and intravenous perfusion started; TIME 0, Peak ischemia time; TIME +1, One hour after reperfusion; TIME +12, Twelve hours after reperfusion; TIME +24, Twenty-four hours after reperfusion; CVMs, cardiovascular medications; rSO2, regional tissue oximetry; CPK, creatine phosphokinase; GSH, glutathione; TEAC, total antioxidant capacity; T0, from peripheral vein 1 hr before flap or recipient vessels were clamped; T1, from flap immediately after arterial clamp was opened; T2, from flap 10 mins after reperfusion; T3, from peripheral vein immediately after venous anastomosis; T4, from peripheral vein 1 hr after venous anastomosis; S1, after dissection of flap before division of vessels; S2, after anastomosis of both veins

Author	Number of Patients/Flaps	Ischemia-Reducing Agent	Ischemia Period (minutes)	Ischemia-Reperfusion Injury (IRI)					
				Flap Loss: TFL, PFL, PFN	Thrombosis: Arterial, Venous, Unspecified	Systemic Response		Vascular Response	
Booi et al. [10]	18/18; TG: 8, CG: 10	Arginine	TG: 54 ± 11, CG: 44 ± 3, p = 1.00	Major PFL: TG: 0, CG: 3; Minor PFL: TG: 2, CG: 2	-	-		Perioperative Zone IV Blood Flow (Perfusion Units): TG: higher, CG: lower, p = 0.04	
La Padula et al. [8]	46/46; TG: 23, CG: 23	Methylprednisolone	TG: 51 ± 10.4, CG: 50 ± 11.3, p = 0.11	TFL: TG: 0, CG: 2; PFN: TG: 1, CG: 8; p = 0.009	Arterial: TG: 5, CG: 4; Venous: TG: 18, CG: 19	Cytokines 5 days post IRI (pg/mL): IL-1 B TG: 27.6 ± 2.1, CG: 54.3 ± 2.2, p = 0.0004; IL-10: TG: 5.11 ± 3.1, CG: 11.24 ± 3.7, p = 0.00044; TNF: TG: 10.2 ± 5.4, CG: 23.2 ± 1.7, p = 0.00011		-	
Del Rio et al. [12]	24/24; TG: 12, CG: 12	Lidocaine	TG: 68.91, CG: 63.25, p = 0.93	PFN: TG: 1, CG: 3	-	MDA (μmol/L) Basal: TG: 1.26, CG: 1.20; Time 0: TG: 1.51, CG: 1.57; Time +1: TG: 1.50, CG: 1.65; Time +12: TG: dec, CG: dec; Time +24: TG: dec, CG: dec	SOD (U/mL) Basal: TG: 78.5, CG: 78.9; Time 0: TG: 92.5, CG: 101.8; Time +1: TG: 96.7, CG: 95.5; Time +12: TG: dec, CG: dec; Time +24: TG: dec, CG: dec	-	
Lee et al. [13]	50/50; Sevoflurane: 25, Propofol: 25	Sevoflurane, Propofol	-	-	-	Syndecan-1, 10 minutes after sitting position: Sevoflurane: 57% increase; Propofol: 21% increase; 1 hour post-op: Sevoflurane: 93% increase; Propofol: 47% increase; p < 0.05		-	
Koolen et al. [16]	482/702; Medication use per flap: Statin: 45, CVM: 70, Statin + CVM: 38, CG: 549	Statins, CMVs	-	TFL: Statin: 0, CVM: 0, Statin + CVM: 0, CG: 12; PFL: Statin: 2, CVM: 2, Statin + CVM: 1, CG: 13; PFN: Statin: 9, CVM: 15, Statin + CVM: 6, CG: 73	-	-		-	
Marre and Hontanilla [17]	182/200	Ischemia time	(By percentile) P25: 63, P50: 79, P75: 89.5, P100: 177	-	Unspecified P50: 14, p = 0.02	-		-	
Coriddi et al. [7]	44/47; Additional venous anastomosis: 9, Intra-op steroids: 33, Post-op steroids: 17; multiple interventions done on some patients	Microvascular compromise management (including additional venous anastomosis); Ischemia-reperfusion management (including steroids)	-	TFL: 13, Completely salvaged flaps: 29, PFL: 5; Additional Venous Anastomosis: TFL: 1, Completely salvaged flaps: 5, PFL: 3 (p = 0.05); Intraoperative steroids: TFL: 9, Completely salvaged flaps: 19, PFL: 5, p = 0.45; Postoperative steroids: TFL: 4, Completely salvaged flaps: 13, PFL: 0, p = 0.20	Arterial: 16, Venous: 30, Both: 1	-		-	
Claroni et al. [11]	54/54; Sevoflurane: 27, Propofol: 27	Sevoflurane, Propofol	-	-	-	CPK (IU/L), end of surgery: Sevoflurane: 133.61 ± 74.07; Propofol: 184.10 ± 100.26; p = 0.04		rSO₂ (%), 4 hours post-op: Sevoflurane: 84.00 ± 8.96; Propofol: 76.16 ± 12.91; p = 0.03	
van den Heuvel et al. [14]	15/17	-	-	PFN: 1	-	Antioxidant concentrations after reperfusion compared to baseline		-	
						GSH: 0.5 h: no significant change (p = 0.328); 1 h: no significant change (p = 0.132); 2 h: increase (p = 0.088); Uric acid: 0.5 h: increase (p = 0.002); 1 h: increase (p = 0.000); 2 h: increase (p = 0.040)	Vitamin E: 0.5 h: increase (p = 0.000); 1 h: increase (p = 0.046); 2 h: no significant change (p = 0.847); TEAC: 0.5 h: no significant change (p = 0.373); 1 h: decrease (p = 0.046); 2 h: increase (p = 0.000)		
Lantieri et al. [15]	20/20	-	48	-	-	-		Endothelin-1 levels, blood samples (pg/mL): T0: 3.87 ± 0.81; T1: 4.34 ± 0.85 (p < 0.0001); T2: 4.5 ± 1.03; T3: 3.79 ± 0.79; T4: 3.83 ± 0.8; increase from baseline (T0)	Revised Table Cell Endothelin-1 levels, tissue samples (pg/mg): S1: 3.8 ± 0.8; S2: 5.2 ± 0.6 (p < 0.0001); increase from S1

Flap loss was the most commonly reported manifestation of IRI, cited in six studies [7,8,10,12,14,16], followed by inflammatory and oxidative stress markers, cited in five studies [8,11,12-14], and then microvascular thrombosis, cited in three studies [7,8,17]. Of the studies that reported flap loss, La Padula et al. [8] demonstrated a significantly lower prevalence of PFN in the group treated with postoperative IV methylprednisolone (2 mg/kg in 250 cc of injectable saline infused over one hour for five days) than in the control group treated with injectable saline (p = 0.0009), while Coriddi et al. [7] retrospectively demonstrated that additional venous anastomosis was a significantly more common intervention in flaps that experienced PFL compared with those that achieved complete salvage or experienced TFL (p = 0.05). Data regarding flap loss are summarized below (TFL: Figure 3; PFL: Figure 4; and PFN: Figure 5). 

**Figure 3:**
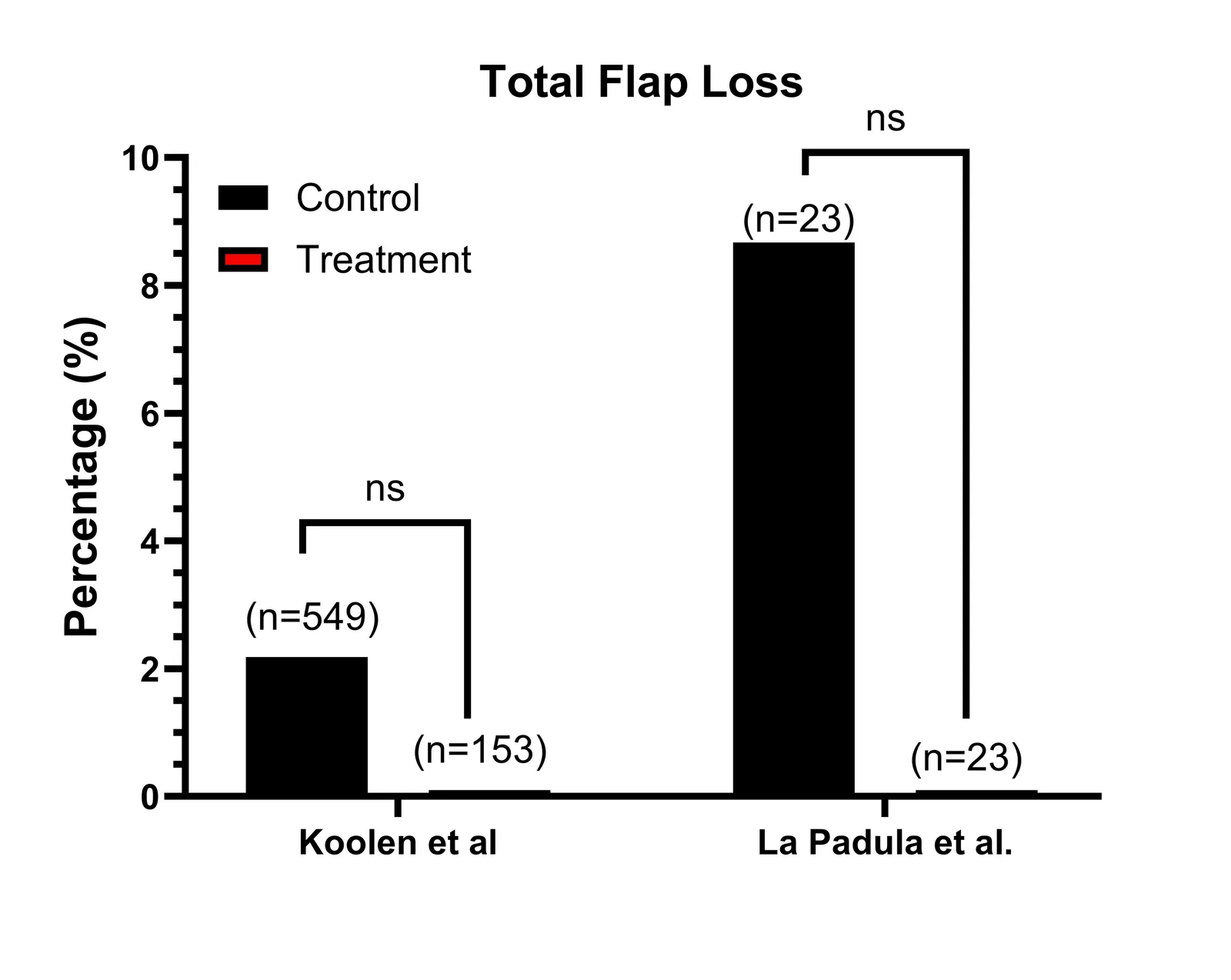
Total Flap Loss (TFL) Bar Chart Reported outcomes of flaps that developed postoperative TFL following mastectomy and breast reconstruction, expressed as a percentage of total flaps per study group, in patients who were treated with various cardiovascular medications (Koolen et al. [16]*) or methylprednisolone (La Padula et al. [8]) versus control groups. *Ischemia-reducing agent data pooled from three medication groups (statins, cardiovascular medications, and statins + cardiovascular medications).

**Figure 4:**
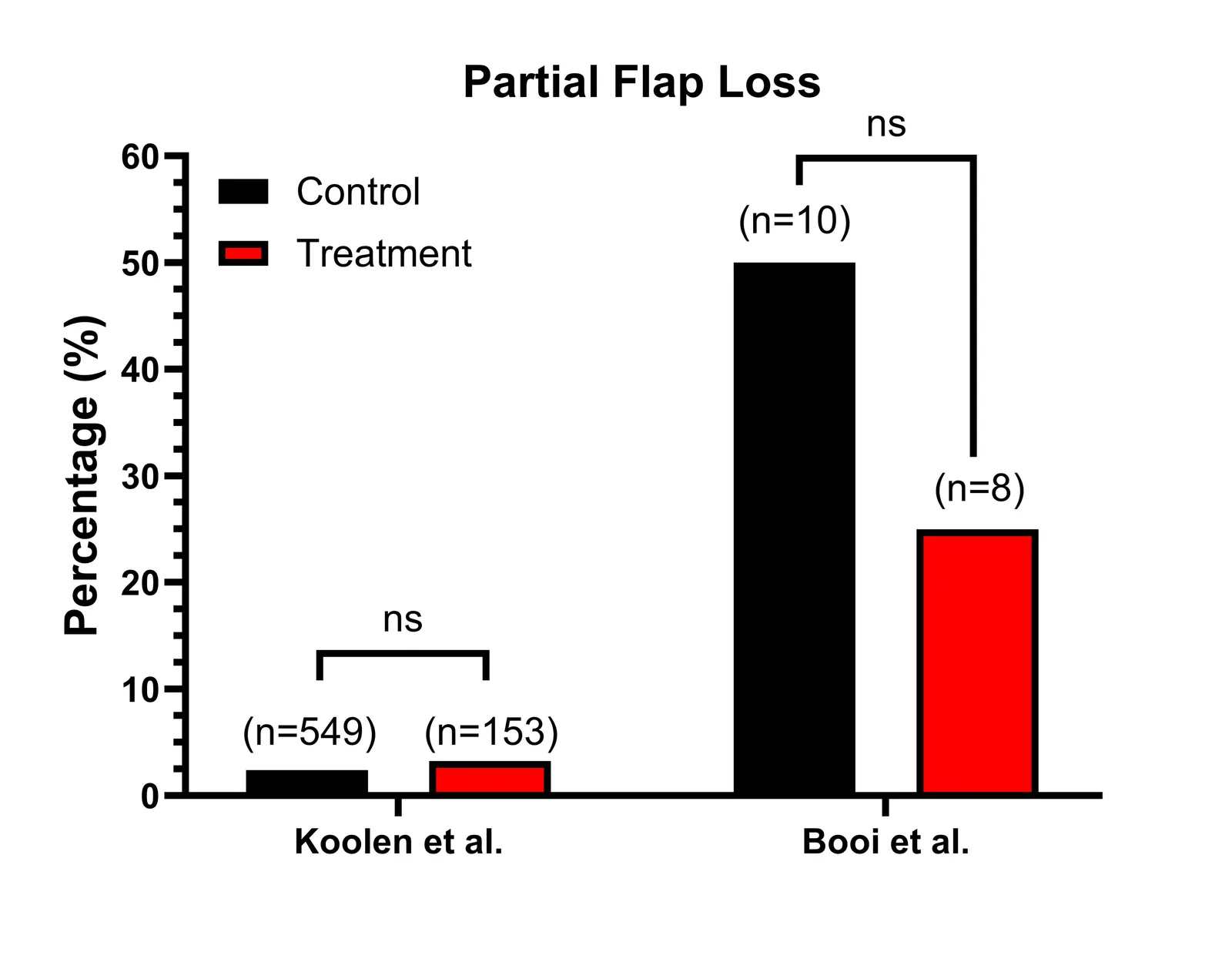
Partial Flap Loss (PFL) Bar Chart Reported outcomes of flaps that developed postoperative PFL following mastectomy and breast reconstruction, expressed as a percentage of total flaps per study group, in patients who were treated with arginine (Booi et al. [10]) or various cardiovascular medications (Koolen et al. [16]*), versus control groups. *Ischemia-reducing agent data pooled from three medication groups (statins, cardiovascular medications, and statins + cardiovascular medications).

**Figure 5:**
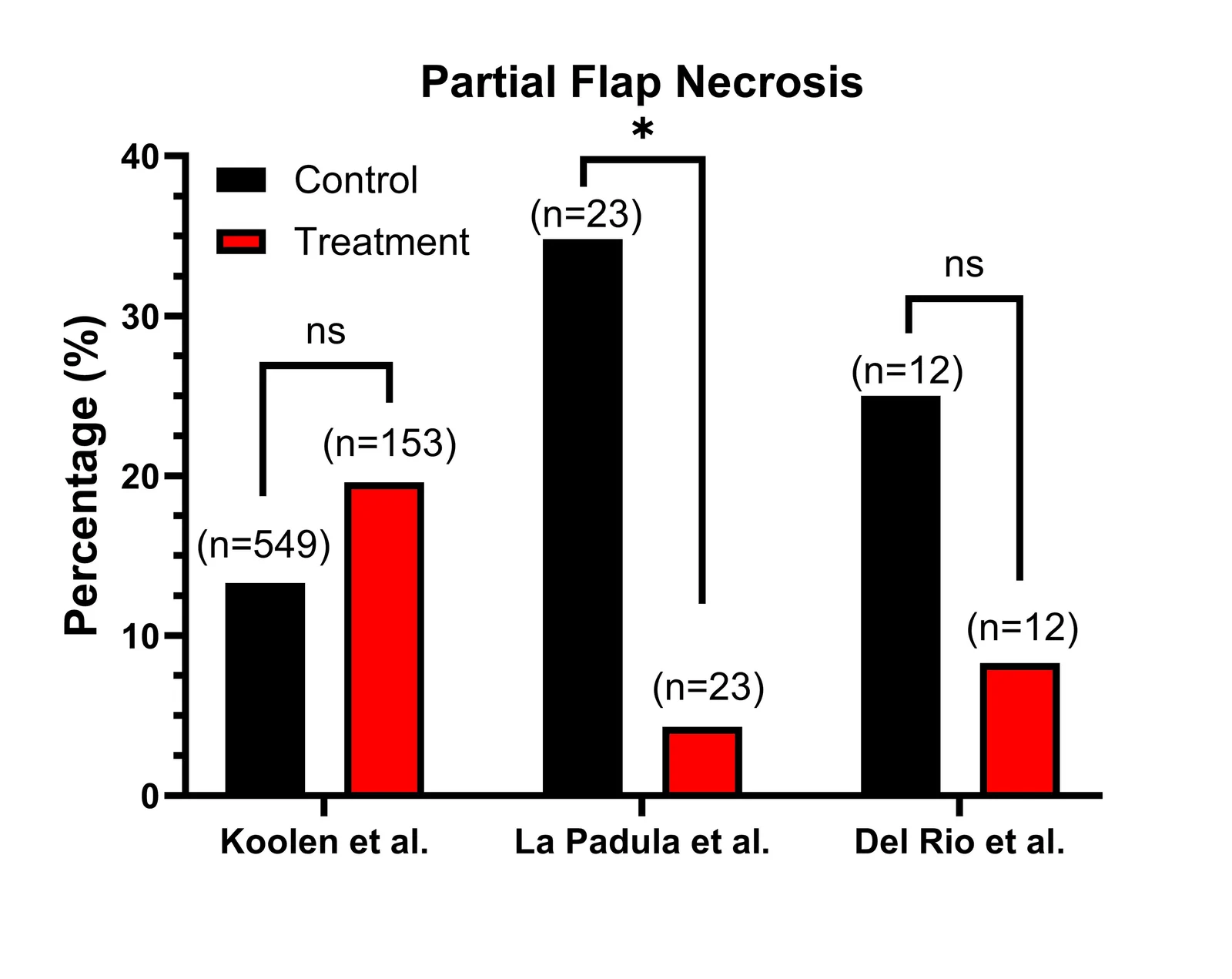
Partial Flap Necrosis (PFN) Bar Chart Reported outcomes of flaps that developed postoperative PFN following mastectomy and breast reconstruction, expressed as a percentage of total flaps per study group, in patients who were treated with lidocaine (Del Rio et al. [12]), various cardiovascular medications (Koolen et al. [16]*), or methylprednisolone† (La Padula et al. [8]), versus control groups. *Ischemia-reducing agent data pooled from three medication groups (statins, cardiovascular medications, and statins + cardiovascular medications); †statistically significant (p = 0.009).

Two non-comparative studies [14,15] observed the effects of IRI on flap tissue, reporting systemic and vascular outcomes. van den Heuvel et al. [14] observed the following significant antioxidant level trends (all compared with baseline): uric acid levels increased at half an hour, one hour, and two hours after reperfusion; vitamin E levels increased at half an hour and one hour after reperfusion only; and total antioxidant capacity (TEAC) decreased at one and two hours after reperfusion [14]. Lantieri et al. [15] observed significantly increased endothelin-1 levels in the blood and tissue of free flaps following reperfusion.

Three studies investigated the effects of different types of anesthesia on IRI. The effect of lidocaine (1.5 mg/kg during anesthesia induction + 2 mg/kg/hour until the end of surgery) on plasma levels of two oxidative stress markers - malondialdehyde (MDA) and SOD - was investigated at different times before, during, and after reperfusion by Del Rio et al. [12]. Although no significant difference was found, data trends showed a greater increase in SOD and a greater decrease in MDA at one hour post-reperfusion in the lidocaine group compared with the control group. These trends did not persist at 12 and 24 hours post-reperfusion [12]. Lee et al. [13] reported the perioperative plasma concentrations of syndecan-1 in patients treated with either sevoflurane-remifentanil anesthesia or propofol-remifentanil anesthesia, finding that the propofol group demonstrated significantly lower concentrations of syndecan-1, both 10 minutes after seating the patients upright during surgery and one hour postoperatively (p < 0.05). Claroni et al. [11] also compared the use of sevoflurane versus propofol and found significantly higher regional tissue oximetry (rSO₂) four hours postoperatively (p = 0.03) and significantly lower creatine phosphokinase (CPK) at the end of surgery (p = 0.04) in the flap tissue of patients treated with sevoflurane. 

Booi et al. [10] reported a significantly higher average level of perioperative microcirculatory blood flow to Zone IV (most distal portion) of the flap in the group given arginine (30 g dissolved in 1000 mL of 0.9% sodium chloride) than in the control group (p = 0.04). This was measured via laser Doppler flowmetry every hour after reperfusion for five hours, yielding a significant difference each time. La Padula et al. [8] reported significantly lower levels of cytokines - IL-1β (p = 0.0004), IL-10 (p = 0.00044), and TNF (p = 0.00011) - in the methylprednisolone group than in the control group five days post-IRI. Furthermore, La Padula et al. [8] noted that complete resolution of IRI based on clinical signs occurred significantly sooner in the treatment group (p = 0.00001).

Discussion

Principle Findings

The most significant finding from this study was that many adjunctive therapies utilized in breast reconstruction following mastectomy reduce instances of flap loss, a specific manifestation of IRI. Other findings include the following: many therapies demonstrated IRI reduction through the analysis of systemic responses (such as inflammation and oxidative stress), and the use of anesthesia as an ischemia-reducing agent requires further research.

Interpretation

Reported reductions in outcomes of flap loss (Table 3) yielded either statistically significant or trending data that suggested the efficacy of certain interventions against IRI. Specific outcomes for flap loss include PFN (tissue death in a specific region of flap tissue), PFL (failure of a specific region of flap tissue to survive, often due to necrosis, thrombosis, and other complications), and complete flap loss (failure of a significant portion or the entire flap tissue to survive). PFL is a notoriously vague term with no standardized scale to assess damage, as previously established by another article in 2013 [18]; therefore, included studies used clinical parameters (capillary refill time, tissue turgor, flap temperature, and flap color) to assess levels of damage. Five studies in this review [7,8,10,12,16] examined the incidence of flap loss to evaluate the efficacy of their respective adjunctive therapies. Comparison of these studies illustrated that the use of methylprednisolone [8], arginine [10], and lidocaine [12] was associated with various degrees of flap loss reduction, although these differences did not reach statistical significance. The use of dexamethasone [7], additional venous anastomosis [7], and CVMs [16] showed no consistent correlation with flap loss (Figures 3-5).

La Padula et al. [8] showed that the perioperative administration of IV methylprednisolone yielded a significant reduction in PFN, suggesting a protective effect of methylprednisolone on flaps experiencing IRI. The addition of methylprednisolone also demonstrated significantly decreased levels of IL-1B, IL-10, and TNF five days post-IRI compared with controls, revealing that the potential mechanism of this therapy may involve the anti-inflammatory ability of corticosteroids to reduce neutrophil activators and chemoattractants, thereby mitigating edema and resultant microvascular damage [8]. Furthermore, this study [8] reported a significantly shorter length of hospital stay in the methylprednisolone group. The other study to investigate the effects of steroids was Coriddi et al. [7], who conducted a retrospective review that found no significant correlation between occurrences of flap loss and administration of intraoperative dexamethasone (6.8 ± 3.3 mg) or postoperative dexamethasone (43.0 ± 22.4 mg). However, the La Padula et al. study had a higher LOE, which should be considered when drawing conclusions about steroid efficacy in IRI management. 

Koolen et al. [16] investigated the correlation between CVM use - statins, other CVMs, and both - versus a control group that took no prescription medications; this review was conducted on the basis that statins and CVMs have mild anticoagulant and anti-inflammatory effects, which may serve to preserve vascular tone. They found a higher percentage of TFL and lower percentages of PFL and PFN, all in the no-medication (control) group compared with the various medication groups [16].

A previous systematic review published in 2022 [19] observed that the use of preventative nitroglycerin ointment, which metabolizes into nitric oxide (NO) - a potent vasodilator and free radical scavenger - significantly reduced flap necrosis in mastectomy. Booi et al. [10] utilized intravenous arginine as an intervention on the basis that arginine - an NO precursor - would potentially stimulate NO production. This study did not yield significant differences in flap loss between the arginine and alanine (control) groups but did report more overall cases of PFL in the control group. Similarly, Del Rio et al. [12] demonstrated a nonsignificant trend toward slightly reduced PFN in their group treated with intravenous lidocaine for its proposed attenuation of oxidative stress. Del Rio et al. [12] found a slightly greater decrease in MDA - a sensitive marker of lipid peroxidation and oxidative tissue damage - and a slightly greater increase in SOD - an antioxidant that removes reactive oxygen species - in the group treated with lidocaine compared with the control group at one hour post-reperfusion. This trend did not persist at later points in the study (12 and 24 hours post-reperfusion), suggesting that lidocaine may offer a slight protective effect against IRI, but that this protection is short-lived [12]. A later study conducted in 2023 [20] observed similar trends in MDA and SOD in skin flaps of mice treated with platelet-rich plasma, a potential avenue for IRI reduction to be explored in human subjects. 

Other outcome variables, particularly markers of vascular response and thrombosis, are also useful in understanding the effects of specific variables on IRI (Table 3). Marre and Hontanilla [17] correlated higher ischemia times during reconstruction surgery with higher rates of microvascular thrombosis. However, this study had a high ROB and an LOE of IV due to a lack of an adequate control group, so this correlation cannot be reliably established. Reported ischemia times and IRI in the more reliable comparative studies [8,10,12] included in this review failed to support this relationship, as higher ischemia times did not correlate with higher incidences of thrombosis or any type of IRI. Perioperative blood flow to the distal flap, the region least perfused and therefore most susceptible to IRI, was significantly higher for five hours after reperfusion in patients treated with IV arginine than in controls, indicating the potential for arginine supplementation to improve flap salvage [10].

There is dissension on the topic of anesthesia in managing IRI in free-flap breast reconstruction. One study from 2015 [21] concluded that restoring flap flow in the case of IRI should be achieved through the maintenance of the same anesthetic principles used during surgery. However, three studies in this review [11-13] used anesthesia as their adjunctive treatment in attempts to identify the ideal choice for IRI mitigation. Syndecan-1 is a core protein in the vascular endothelial glycocalyx and, therefore, the amount of shedding of syndecan-1 is a marker of endothelial damage due to surgery and IRI [13]. Plasma concentrations of syndecan-1 in patients treated with propofol anesthesia were lower than in patients treated with sevoflurane anesthesia, both during and after breast reconstruction, indicating that propofol may have some protective effect against endothelial injury [13]. However, Claroni et al. reported significantly lower concentrations of CPK and higher regional tissue oximetry postoperatively in patients treated with sevoflurane compared with propofol, suggesting that sevoflurane may confer less inflammation and more perfusion to flap tissue and is therefore the better adjunctive anesthesia choice for IRI management [11]. It is clear that no consensus has been reached on which type of anesthesia is most beneficial for reducing IRI due to either nonsignificant data (as elaborated upon above) [12] or discrepancies between final results [11,13]. 

Two studies in this review were noncomparative studies with moderate ROB and levels of evidence of III [14] and IV [15], so their results are included to bolster the understanding of IRI in breast reconstruction following mastectomy and provide insight into future treatment targets (refer to Table 3). van den Heuvel et al. [14] demonstrated a significant decrease in hydrophilic antioxidant capacity in flaps experiencing IRI. Lantieri et al. [15] demonstrated significantly increased levels of blood and tissue endothelin-1 during reperfusion, establishing endothelial damage as a key factor in IRI.

Due to their ability to increase the rate of postoperative complications, comorbidities must be accounted for when assessing outcomes of flap complications. A prior systematic review and meta-analysis [22] found that diabetic women who underwent breast reconstruction suffered significantly higher rates of flap necrosis. Eight of the studies in this review [7,8,10-13,16,17] disclosed risk factors in their patient populations (Table 2). Of these eight, seven [7,8,10-13,17] found no significant difference in rates of comorbidities between groups, which, along with similarity of demographic factors such as age and BMI, allowed investigators to establish baseline equivalence of study groups and therefore lower the ROB in establishing a correlation between their ischemia-reducing agent and observed outcome. One study [16] reported a significant difference in rates of hypertension, diabetes, and hyperlipidemia, as well as significant differences in complication rates between groups, all of which contributed to this study's score of “0” for the “baseline equivalence of groups” criterion in the MINORS score (Figure 2). The study [16] acknowledges this nonequivalence as a limitation, attributing the higher rates of medication use in individuals with comorbidities to the management of their pre-existing conditions. The majority of studies in this review demonstrated a lack of confounding variables in the interpretation of their study results.

Limitations

The findings of this study must be considered in the context of its limitations. Many of the studies in this review had small sample sizes, negatively impacting their statistical power and reducing the generalizability of the results. Another limitation was the wide variability in treatment interventions and measurements of IRI across studies. This made it difficult to establish strong direct comparisons and was the reason our study was not eligible for meta-analysis.

Implications

The findings of this review have potential implications for the perioperative management of patients undergoing autologous breast reconstruction. Although the available evidence does not establish a single optimal adjunctive therapy for IRI, several interventions demonstrated potentially beneficial effects on flap perfusion, necrosis, inflammatory markers, or oxidative stress. In particular, perioperative corticosteroid administration was associated with reduced PFN and inflammatory markers, while intravenous arginine was associated with improved distal flap perfusion. However, heterogeneity in interventions and IRI outcome measures, as well as limited sample sizes, precludes definitive recommendations regarding the routine use of these therapies. The findings therefore support continued investigation of adjunctive strategies that may reduce IRI and improve free-flap outcomes.

Future research

Areas for future research on adjunctive treatments for IRI following mastectomy include anesthesia, given the lack of consensus regarding the anesthetic approach that best mitigates IRI, and hydrophilic antioxidant supplementation, given the observed decrease in hydrophilic antioxidant capacity in flaps experiencing IRI. Furthermore, future studies should investigate outcomes of IRI-reducing interventions across populations with various risk factors to better understand the interplay between these treatments and pre-existing comorbidities. We hope that the detailed presentation of information and comparison of key variables in this review will facilitate the work of future surgeons and investigators seeking to improve IRI outcomes in patients undergoing post-mastectomy breast reconstruction.

## Conclusions

The use of adjunctive therapies is associated with reduced rates of IRI in breast reconstruction following mastectomy. Postoperative infusion of IV arginine resulted in greater blood flow to the distal flap. Perioperative IV methylprednisolone has shown promising results, given the significantly shorter length of hospital stay, lower concentrations of cytokines following IRI, and reduced rates of PFN observed in the treatment group versus the control group. Larger studies should be conducted to solidify these results. Future research should focus on steroid and/or vasodilator administration as potential treatment avenues, reconcile discrepancies in the literature regarding the anesthetic approach associated with reduced IRI, and seek to establish lasting protective effects against IRI in flap tissue.
